# A Novel Ammonium Carboxylate Salt of Undecylenic Acid for the Topical Treatment of Gram‐Positive and Antibiotic‐Resistant Skin Infections

**DOI:** 10.1111/exd.70075

**Published:** 2025-03-10

**Authors:** Alyce Mayfosh, Thomas Rau

**Affiliations:** ^1^ Wintermute Biomedical Inc. Missoula Montana USA

**Keywords:** antibiotic resistance, fatty acids, gram‐positive, impetigo, infection, MRSA, undecylenic acid

## Abstract

Uncomplicated topical skin infections like impetigo, caused by gram‐positive bacteria such as *Staphylococcus aureus* and *Streptococcus pyogenes* , are a common global health issue, particularly affecting children. With increasing antimicrobial resistance, conventional treatments such as mupirocin are becoming ineffective, highlighting the necessity for new antimicrobial development. Fatty acids have long shown potential as novel antimicrobials, but their development has been limited by solubility and efficacy concerns in topical applications. We previously discovered that combining the amino acid L‐arginine with an 11‐carbon fatty acid, undecylenic acid, produced a water‐soluble ammonium carboxylate salt, arginine undecylenate, referred to as GS‐1, that elicits potent antimicrobial activity. Under CLSI test conditions, GS‐1 showed effective antibacterial activity against clinical isolates of methicillin‐sensitive *S. aureus* (MSSA), methicillin‐resistant *S. aureus* (MRSA), vancomycin‐intermediate *S. aureus* , and *S. pyogenes* , with MICs of 0.60–1.26 mg/mL and MBCs of 0.63–5.04 mg/mL, respectively. Fluorescence microscopy revealed GS‐1 to elicit antibacterial activity by rapidly permeabilising bacterial membranes and inducing reactive oxygen species formation. Serial exposure of 5 MRSA clinical isolates to sub‐lethal doses of GS‐1 did not appear to induce resistance. In fact, compared to mupirocin, repeated exposures to GS‐1 appeared to sensitise bacteria to GS‐1. In an animal model of skin infection, topical GS‐1 successfully eradicated MRSA from infected, abraded skin after 6 days of treatment with no signs of toxicity. Finally, repeated topical GS‐1 exposure in humans caused no irritation or sensitisation. These results support GS‐1 as a potential novel topical antibacterial for the treatment of impetigo and other skin infections.

## 1 Introduction

Uncomplicated topical skin infections caused by gram‐positive cocci (impetigo) remain a common healthcare issue that affects over 140 million patients worldwide, with the vast majority of them being children between the ages of three and 12 [1]. Impetigo involves methicillin‐sensitive *Staphylococcus aureus* (MSSA), methicillin‐resistant *Staphylococcus aureus* (MRSA) and *Streptococcus pyogenes* (Group A *streptococcus*) [2]. The primary treatment for uncomplicated skin infections and decolonisation of MRSA is mupirocin (Bactroban). While topically effective at high concentrations (2% w/w), the primary causative agents of impetigo (MSSA, MRSA) have shown the ability to rapidly develop resistance to mupirocin [3, 4]. Global prevalence of mupirocin resistance in *S. aureus* and MRSA is approximately 7%–14%, with increases in high‐level resistance (> 512 μg/mL) observed in recent years [5]. In fact, with the widespread overuse of antimicrobials, the gram‐positive strains that produce skin and soft tissue infections are developing resistance to many conventional, commercially available treatments [6], thus necessitating the development of new antimicrobial agents.

Antimicrobial fatty acids represent a known but relatively underutilised resource in the treatment of bacterial infections of the skin and underlying tissue. Fatty acids are found commonly in nature and are key elements in both prokaryotic and eukaryotic cells. While the role of fatty acids in cell structure and metabolism is well established, the potential of fatty acids as antimicrobial agents, particularly in topical applications, is less well understood. A wide range of fatty acids has been found to exert broad‐spectrum activity against a range of common gram‐positive and gram‐negative pathogenic bacteria [7, 8, 9, 10]. There are several advantages to utilising fatty acids in the treatment of microbial pathogens: they do not appear to quickly develop resistance [11], and several are naturally occurring skin components in the human innate immune response to pathogens [12, 13], and as such, they have extremely low toxicity risks associated with their use.

While there are clear advantages in the utilisation of fatty acids as antibacterial agents, there are several issues regarding their practical application. Physically, fatty acids are almost completely water insoluble and must be combined with toxic solubilising agents, dissolved in organic solvents, or chemically modified (esterified) to be effectively utilised in practical applications. In certain cases, these modifications (e.g. methyl esterification) reduce the antimicrobial efficacy of fatty acids when compared to their unmodified forms [14]. In an attempt to overcome these issues, we previously developed a novel compound utilising undecylenic acid, an 11‐carbon unsaturated fatty acid, combined with an essential amino acid, L‐arginine, to produce a stable water‐soluble ammonium carboxylate salt with broad spectrum antibacterial effects. While there is clear evidence‐based research on the antifungal effects of undecylenic acid [15] there is scant research demonstrating any type of antibacterial effects.

This study outlines the investigation of a stable, water‐soluble solution of undecylenic acid, referred to here as GS‐1, as a novel impetigo therapy. Here, we show that GS‐1 produces MICs and MBCs in clinical isolates of MRSA, MSSA, *S. pyogenes* , and vancomycin‐intermediate *S. aureus* (VISA). We demonstrate that GS‐1 induces bactericidal activity by initiating rapid membrane permeabilisation and reactive oxygen species (ROS) production. Further, we demonstrate that GS‐1 does not appear to induce resistance in impetigo‐causing bacteria following multiple exposures. Finally, we show that topical GS‐1 is an effective and safe antibacterial in a rodent model of abraded skin infection, and that repeated exposure to topical GS‐1 is well tolerated in human subjects.

## 2 Materials and Methods

### 2.1 Clinical Isolates

Clinical isolates of MRSA, MSSA, and VISA were isolated from hospital patients with bacteremia (via blood cultures), urinary tract infections (via loop urine culture and streak plating on 5% sheep blood agar and Maconkey agar), or infected wounds (via sterile swabs of infected tissue and streak plating on 5% sheep blood agar), and verified via sequencing (isolates generously donated from Kalispell Regional Medical Center, Montana USA). *S. pyogenes* (group A streptococcus) isolates were obtained from the Peter Doherty Institute for Infection and Immunity, Victoria, Australia. All clinical isolates were stored in litmus milk at −20°C prior to testing.

### 2.2 Bacterial Culture

Clinical isolates were cultured on tryptic soy agar (TSA) with 5% sheep's blood for 24 h prior to testing. Organism suspensions were then prepared by taking single colonies and growing them to log‐phase for 4–6 h in Mueller–Hinton broth for MRSA, MSSA, and VISA isolates (Thermo Fisher, Australia), or Oxoid Todd‐Hewitt Broth (Thermo Fisher, Australia) supplemented with Oxoid Yeast Extract (Thermo Fisher, Australia) for *S. pyogenes* isolates. Isolates were then further diluted to an optical density equivalent to 1 × 10^6^ CFU/mL using a Grant bio DEN‐1 densitometer (Grant Instruments, USA) in their respective media as above.

### 2.3 Determination of MIC and MBC

The MIC and MBC of GS‐1 were assessed against clinical isolates of MRSA, MSSA, VISA, and *S. pyogenes* using a 96‐well microplate format under Clinical Laboratory Standards Institute (CLSI) M26‐A guidelines [16].

Suspensions of isolates were prepared at 1 × 10^5^ CFU/mL in Oxoid Iso‐Sensitest Broth (Thermo Fisher, Australia) for MRSA, MSSA, and VISA isolates or Oxoid Todd‐Hewitt Broth with yeast extract for *S. pyogenes* isolates.

Serial dilutions of GS‐1 were generated in the appropriate media respective to the clinical isolates, and 100 μL of each dilution was added to 96‐well plates in triplicate. Bacterial isolate suspensions (100 μL) were added to wells containing GS‐1 or media only. Plates were then incubated for 24 h at 37°C with 5% CO_2_. MIC was determined by visual assessment, with no turbidity indicating growth inhibition.

Technical replicates of the GS‐1‐treated isolates at the MIC concentration and 3 concentrations above were pooled, and bacteria pelleted at 7000 rpm for 15 min. The supernatant was removed, and the pellet was resuspended in 1 mL sterile saline. Treated isolates were serially diluted, and 100 μL of each solution was plated onto TSA plates with 5% sheep's blood and incubated for 24 h at 37°C.

For growth and contamination controls, media‐only and water‐only controls were set up in the 96‐well plate and processed consistently with experimental samples to ensure sterility and quality control. Positive control wells containing bacteria, media and sterile water were diluted, plated, and counted to determine the amount of growth from the starting inoculum and to ensure robust growth of the test organisms.

Following incubation, the number of colonies was counted. The MBC for each isolate was determined as the concentration of GS‐1 that resulted in a 99.99% reduction in bacteria (CFU/mL) from the starting inoculum.

### 2.4 MRSA Time‐Kill Assay

Five clinical isolates of MRSA were prepared at 1 × 10^6^ CFU/mL in Oxoid Sensitest Broth, and 100 μL added to wells in a 96‐well plate in triplicate containing 100 μL GS‐1 (final concentration 1.26 mg/mL) or media only. Samples were extracted at 0, 2, 4, 12, and 24 h, triplicates pooled, bacteria pelleted, resuspended in 1 mL sterile saline, and 100 μL plated in a dilution series on TSA with 5% sheep's blood, incubated for 24 h at 37°C, and colonies counted to determine CFU/mL.

### 2.5 PI, SYTO 9, and DHE Staining

*S. aureus* (ATCC Strain BAA‐1026) cultures were grown overnight in Mueller‐Hinton broth until cell density reached 1.8 × 10^9^ CFU/mL. Then, 900 μL of this bacterial suspension was transferred to sterile Eppendorf tubes. Either sterile water or GS‐1 at a final concentration of 1.26 mg/mL was added to the bacterial suspensions and incubated at 37°C.

15 min before imaging, cells were stained with either 3 μL PI (Thermo Fisher Scientific, Waltham, MA, USA), 3 μL of SYTO 9 (Thermo Fisher Scientific) or 3 μL of DHE at 10 μM (Sigma‐Aldrich, Burlington, MA, USA). At 15 min, 1, and 2 h, each tube was vortexed for 15 s, and 15 μL removed and placed onto a glass slide, covered with a coverslip, and cells imaged immediately on a Leica DM IL LED Microscope (Leica, USA).

Fluorescence was quantified by calculating the fluorescent area fraction (PI) and fluorescence intensity (SYTO 9 and DHE) using ImageJ software (NIH, USA).

### 2.6 Repeated Exposure to GS‐1

Five clinical isolates of MRSA were prepared at 1 × 10^6^ CFU/mL and 100 μL seeded in 96‐well plates containing 100 μL GS‐1 (final concentration 616 or 308 μg/mL) and incubated at 37°C with 5% CO_2_. Every 24 h, samples were extracted, triplicates pooled, pelleted, and either resuspended in 300 μL Mueller‐Hinton media and re‐added to the 96‐well plate containing GS‐1 (final concentration 616 or 308 μg/mL), or serial dilutions were created, 100 μL plated onto TSA with 5% sheep's blood, incubated for 24 h at 37°C, and colonies counted to determine CFU/mL. This was repeated for a total of 25 day.

After 24 day, naïve MRSA isolates and isolates previously exposed to GS‐1 as above (1 × 10^6^ CFU/mL) were treated with GS‐1 at a final concentration of 308 μg/mL in triplicate for 24 h. Following incubation, triplicates were pooled, pelleted, resuspended in sterile saline, and 100 μL plated in dilution series on TSA with 5% sheep's blood, incubated for 24 h at 37°C and colonies counted to determine CFU/mL.

Twelve MRSA isolates were prepared at 8.1 × 10^7^ CFU/mL, 100 μL seeded in 96‐well plates in triplicate, and combined with 100 μL of either saline, mupirocin (final concentration 2.5 mg/mL), or GS‐1 (final concentration 3.1 mg/mL) for 7 day. Bacteria were re‐exposed to GS‐1 or mupirocin, and bacterial growth (CFU/mL) measured every 24 h as above.

### 2.7 Animal Model of MRSA Skin Infection

All animal handling and treatment was approved by the University of Montana Institutional Animal Care and Use Committee (052‐18TRPC‐103 118; approved 31 October 2018). Animals were given free access to food, water, and enrichment. Animals were co‐housed for a 72‐h acclimation period prior to the beginning of experiments and then housed singly during the dosing protocol.

Sixteen male Sprague–Dawley rats were lightly anaesthetised using 3%–4% isoflurane, and a 4 × 4 cm area directly between the shoulder blades was shaved and cleaned with betadine and alcohol. Under anaesthesia, the skin was abraded with a #10 sterile scalpel blade.

Twenty‐four hours before animal infection, bacterial cultures of eight MRSA isolates were sub‐cultured onto TSA with 5% sheep's blood and incubated at 37°C. After 24 h of incubation, colonies were collected from the culture plate and a 3 × 10^8^ CFU/mL suspension was made in sterile water. This was subsequently diluted into sterile saline to 1 × 10^6^ CFU in 50 μL of saline.

Immediately after skin abrasion, MRSA isolates (1 × 10^6^ CFU in 50 μL of saline) were rubbed vigorously into the abraded skin for 30 s with a sterile plastic spatula and allowed to dry on the skin for 5 min. Two rats were inoculated per MRSA isolate. Twenty‐four hours post‐infection, a topical swab was taken and plated onto TSA with 5% sheeps blood to confirm an active infection.

Following confirmation of infection, rats were randomly assigned to receive either GS‐1 (157.6 mg/mL) or saline treatment, with one rat in each group for every MRSA isolate. GS‐1 and saline (100 μL) treatments were administered topically using a sterile plastic spatula for even spreading across the application site, twice daily, 8 h apart, for 6 day.

On day 7, rats were euthanised and the 4 × 4 cm infected area was swabbed and plated on 5% SBA plates to determine the presence of topical infection. The area was then cleaned with betadine and alcohol, and two tissue punches were taken from the skin of each animal, each with a diameter of 4 mm and a depth of 6 mm, and homogenised together. Samples were then diluted in sterile saline and 100 μL plated onto TSA with 5% sheep's blood, incubated for 24 h at 37°C, 5% CO_2_. Bacterial counts were performed to calculate CFU. A 10 mL blood draw was taken from each rat via cardiac puncture and clinical chemistry and haematology were analysed.

### 2.8 Human Repeat Insult Patch Test

A Repeat Insult Patch Test (RIPT) was conducted in accordance with the ethical principles outlined in the Declaration of Helsinki and adhered to International Council for Harmonisation Good Clinical Practice (ICH GCP) standards.

The RIPT was performed at Eurofins | CRL Inc., an independent clinical research laboratory with standardised procedures designed to ensure participant safety and data reliability.

All study participants provided written informed consent before participation. The study protocol was reviewed and conducted under the Standard Operating Procedures (SOPs) of Eurofins | CRL Inc., which aligns with industry best practices and applicable regulatory guidelines for cosmetic and topical product testing.

As the study involved topical application of a well‐characterised test material (GS‐1; undecylenic acid and arginine) with minimal risk, did not involve investigational drugs or medical devices, and did not collect identifiable health information, it did not meet the criteria for human subjects research requiring Institutional Review Board (IRB) approval under 45 CFR 46.102. No adverse events were reported, and all data were collected in compliance with confidentiality and ethical standards. A total of 120 male and female participants aged 18–70 years, meeting inclusion and exclusion criteria, were enrolled in the study. Of these, 111 subjects completed the study, including 57 with self‐perceived sensitive skin.

The Induction Phase consisted of nine applications of GS‐1 over 3 weeks, with patches applied to the same site three times per week. GS‐1 at 154 mg/mL (150 μL) was applied to occlusive patches consisting of a 2 × 2 cm fabric area, which were affixed to marked test sites on the back of each subject. Test sites were cleansed with 70% isopropyl alcohol before each application. Participants removed the patches after 24 h and returned for dermal evaluations prior to the next application. Rest periods included 24 h between weekday applications and 48 h over weekends.

Following a 10–21 day rest period after the Induction Phase, subjects underwent a Challenge Phase. In this phase, a challenge patch containing GS‐1 at 154 mg/mL (150 μL) was applied to a virgin site on the back. After 24 h, patches were removed, and test sites were evaluated at 24, 48, 72, and 96 h post‐application for dermal reactions. Dermal reactions were assessed using a standardised dermal scoring system for erythema, oedema, and other dermal reactions.

### 2.9 Statistics

Statistical analyses (mean, median, standard deviation, confidence intervals, two‐tailed *t*‐test, Mann–Whitney *U* test, two‐way ANOVA) were performed with GraphPad Prism 9.0 software (GraphPad Software, San Diego, CA, USA).

## 3 Results

### 3.1 Antibacterial Activity of GS‐1 In Vitro

Undecylenic acid is an 11‐carbon monounsaturated fatty acid with known antifungal and anticancer activity [17]. Characterisation of this undecylenic acid‐arginine salt (previously referred to as GS‐1) has been described previously, whereby NMR and GCMS confirmed that combining undecylenic acid and arginine did not form a new chemical entity [17]. TEM revealed the formation of vesicle‐like structures [17]. In this study, stability testing of GS‐1 was conducted over 12 months at room temperature (25°C, 60% RH) and accelerated conditions (40°C, 75% RH), with analytical and physical test results demonstrating that this formulation of undecylenic acid remains stable for at least 12 months (Table S1).

**FIGURE 1:**
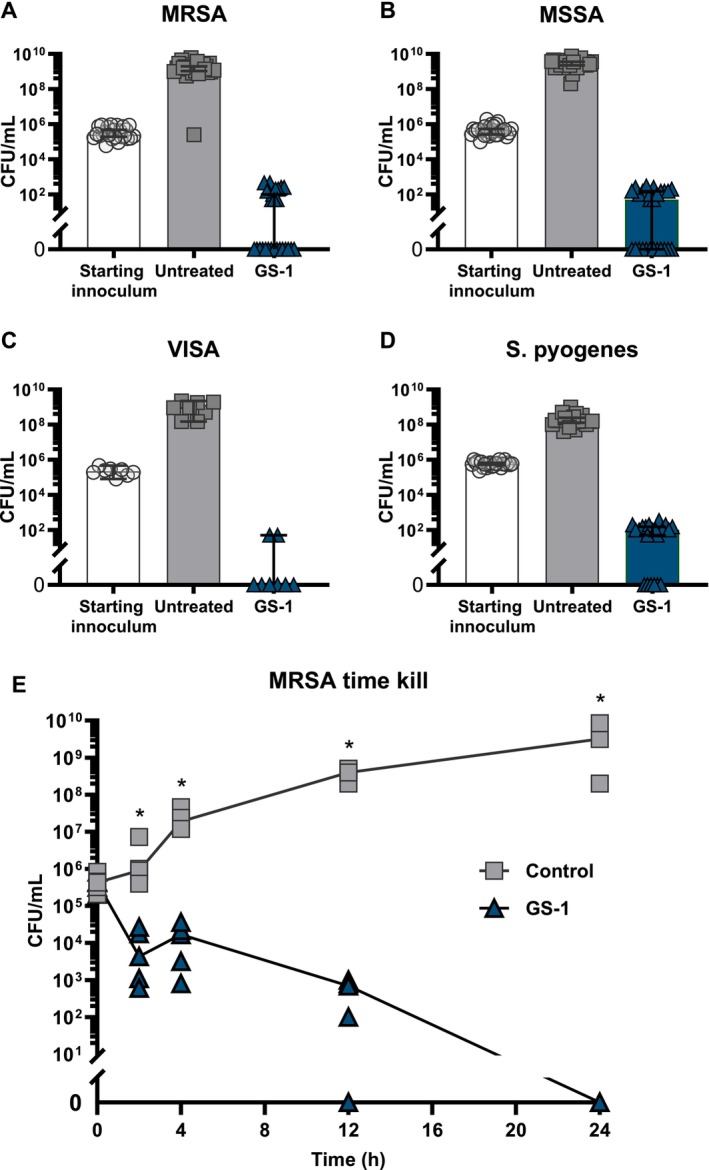
GS‐1 displays activity against gram‐positive pathogens in vitro. (A) Twenty‐six Clinical isolates of MRSA were treated with GS‐1 at 1.26–5.04 mg/mL for 24 h. (B) Twenty‐five clinical isolates of MSSA were treated with GS‐1 at 1.26–2.52 mg/mL for 24 h. (C) Eight clinical isolates of VISA were treated with GS‐1 at 1.2–2.4 mg/mL for 24 h. (D) Twenty‐three clinical isolates of *S. pyogenes* were treated with GS‐1 at 0.63–1.26 mg/mL for 24 h. (E) Five clinical isolates of MRSA were exposed to GS‐1 at 1.26 mg/mL for 24 h. Samples were taken, diluted, plated, and counted to determine the amount of bacteria recovered at the time points indicated (median ± 95% CI, (E) multiple Mann–Whitney *U* tests with Bonferroni correction. *p < 0.05).

Uncomplicated skin infections are mostly mediated by gram‐positive bacteria. To assess the activity of GS‐1 against common impetigo‐causing bacteria, the fatty acid conjugate was tested against clinical isolates of MRSA, MSSA, VISA, and *S. pyogenes* under CLSI guidelines, and MICs and MBCs were determined. Testing GS‐1 against 26 clinical isolates of MRSA revealed MBC_100_ was achieved at 1.26 mg/mL in 7 isolates, 2.52 mg/mL in 16 isolates, and 5.04 mg/mL in 3 isolates. MIC_100_ was achieved at 0.63 mg/mL in 8 isolates and 1.26 mg/mL in 18 isolates (Table 1 and Figure 1A). A similar effect was observed against MSSA with an MIC_100_ at 0.486 mg/mL and MBC_100_ at 0.971 mg/mL (Table 1 and Figure 1B). Eight isolates of VISA demonstrated similar MIC sensitivity to MRSA with an MIC_100_ of 0.500 mg/mL but greater bactericidal sensitivity with an MBC_100_ at 0.770 mg/mL (Table 1 and Figure 1C). *S. pyogenes* proved to be the most sensitive to GS‐1 with an MIC_100_ of 0.335 mg/mL and an MBC_100_ of 0.509 mg/mL (Table 1 and Figure 1D).

**TABLE 1:** MIC_100_ and MBC_100_ of GS‐1 against clinical isolates of impetigo‐causing pathogens.

Species (total number of isolates)	MIC_100_ (number of isolates)	MBC_100_ (number of isolates)
MRSA (26)	0.63 mg/mL (8/26) 1.26 mg/mL (18/26)	1.26 mg/mL (7/26) 2.52 mg/mL (16/26) 5.04 mg/mL (3/26)
MSSA (25)	0.63 mg/mL (25/25)	1.26 mg/mL (15/25) 2.52 mg/mL (10/25)
VISA (8)	0.60 mg/mL (3/8) 1.20 mg/mL (5/8)	1.20 mg/mL (7/8) 2.40 mg/mL (1/8)
*S. pyogenes* (23)	0.63 mg/mL (21/23) 1.26 mg/mL (2/23)	0.63 mg/mL (8/23) 1.26 mg/mL (15/23)

In a time‐kill study against 5 clinical isolates of MRSA, GS‐1 at 1.26 mg/mL reduced viable MRSA concentrations by 97.2% at 2 h (Figure 1E), suggesting rapid activity. By 12 h, CFU was reduced by 2‐log (99%), and 100% was confirmed at 24 h (Figure 1E).

Interestingly, GS‐1 also demonstrated inhibitory activity against several gram‐negative bacterial pathogens (Table S2) and several fungal pathogens (Table S3), although GS‐1 appeared to be more potent against gram‐positive bacteria than gram‐negative bacteria.

### 3.2 GS‐1 Induces Bactericidal Activity by Permeabilising Bacterial Membranes and Inducing Reactive Oxygen Species

**FIGURE 2:**
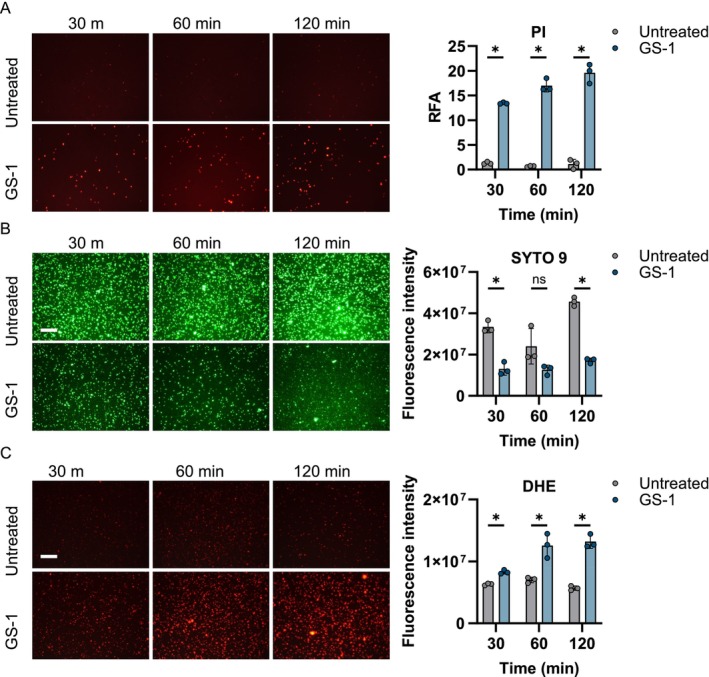
GS‐1 causes membrane permeabilization and ROS production in *S. aureus* . *S. aureus* was treated with 1.26 mg/mL GS‐1 for 30 m, 1 h, and 2 h before staining with (A) PI, (B) SYTO 9, or (C) DHE, and microscopy images were taken at 20× magnification. Images are representative of triplicate images. Graphs represent Relative Fluorescent Area (RFA) or fluorescence intensity (mean ± SD, (A–C), Multiple *t*‐tests with Holm‐Šídák corrections. *p < 0.05).

To understand the mechanism of how GS‐1 induced bacterial cell death, we treated *S. aureus* with GS‐1 and then stained with PI to visualise membrane permeabilization. Within 30 min, PI staining had increased compared to the untreated control, further increasing slightly after 1 h, and modestly increasing further after 2 h (Figure 2A). This suggests membrane permeabilization results from GS‐1 treatment.

To complement PI staining, we treated *S. aureus* with GS‐1, then stained with SYTO 9, a nucleic acid stain that selectively stains viable cells. SYTO 9 staining of *S. aureus* reduced within 30 min of GS‐1 treatment, suggesting a loss in cell viability (Figure 2B), consistent with findings from the PI staining. By 2 h there was a further loss in SYTO 9 signal (Figure 2B), together suggesting GS‐1 induces rapid cell death.

We sought to determine whether GS‐1 caused membrane permeabilisation via the production of reactive oxygen species (ROS). To test this, *S. aureus* was treated with GS‐1 and stained with DHE, a superoxide indicator. Within 30 min of GS‐1 treatment, DHE staining had increased above that in the untreated control, increased at 1 h, and was sustained at 2 h (Figure 2C). This suggests GS‐1 treatment induces ROS production. Given the rapid membrane permeabilisation indicated by the PI staining, it is likely that ROS production occurs downstream of membrane permeabilisation.

Together, these results suggest GS‐1 causes rapid bacterial cell death by permeabilising the bacterial membrane, which in turn causes toxic ROS production.

### 3.3 MRSA Repeatedly Exposed to GS‐1 Did Not Develop Resistance

**FIGURE 3:**
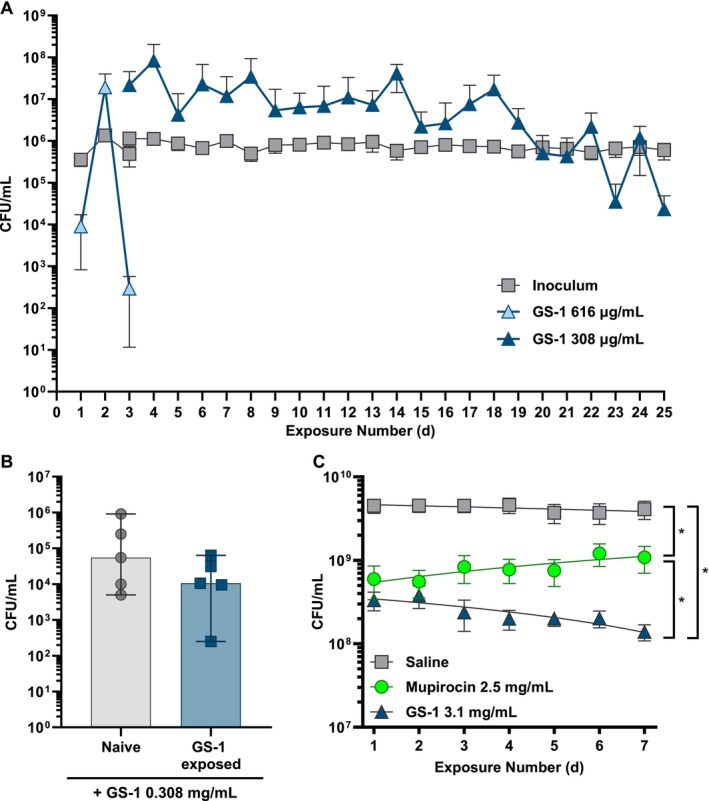
Resistance in MRSA was not observed following repeated exposures to GS‐1. (A) Five clinical isolates of MRSA (1 × 10^6^ CFU/mL) were exposed to GS‐1 25 times. The first three exposures were performed with 0.616 mg/mL, then reduced to 0.308 mg/mL for the remainder of the experiment. CFU/mL was calculated every 24 h. (B) Naïve and GS‐1‐exposed MRSA isolates (from A, 24 cycles; 1 × 10^6^ CFU/mL) were treated with GS‐1 at 0.308 mg/mL for 24 h and CFU/mL determined. (C) Twelve MRSA isolates (8.1 × 10^7^ CFU/mL) were exposed to either saline, mupirocin (2.5 mg/mL) or GS‐1 (3.1 mg/mL) every 24 h for 7 day. Bacterial growth (CFU/mL) was measured every 24 h. Curve represents nonlinear line of best fit (two‐way ANOVA with Turkey's multiple comparisons test. *p < 0.05). (A, B) *n* = 5 clinical isolates; (C), *n* = 12 clinical isolates. (A, C), mean ± SD. (B), median ± 95% CI.

To understand the possible development of resistance to GS‐1, we utilised five clinical MRSA isolates and exposed them to GS‐1 at sub‐MIC for 25 repetitions. Initial treatment began with 0.616 mg/mL GS‐1, but this dose was decreased to 0.308 mg/mL due to low recovery after the third exposure (Figure 3A). For the remaining 21 exposures, dosing was maintained at 0.308 mg/mL. The loss of viability appeared to vary over the study, with recovery counts going up and down by approximately 1 log between exposures. Surprisingly, over the course of this study, bacterial growth rates trended downwards with increasing exposures, with approximately 1 log difference between median levels recovered after treatment of naïve bacteria and those that had been exposed 24 times previously (Figure 3A,B). The bacteria previously exposed to GS‐1 appeared more susceptible to GS‐1 treatment when compared to the naïve bacteria, although this was not statistically significant. Most interestingly, the bacterial levels recovered following repeated exposures to GS‐1 were not higher than naïve bacteria, indicating that the bacteria did not appear to develop resistance to GS‐1.

To confirm the absence of resistance development, we ran repeated exposures against 12 clinical isolates of MRSA alongside mupirocin, the gold‐standard topical antibiotic for bacterial skin infections, such as impetigo. MRSA at a starting concentration of 8.2 × 10^7^ CFU/mL was incubated for 24 h in the presence of mupirocin (2.5 mg/mL), GS‐1 (3.1 mg/mL) or saline. After 24 h of incubation, the number of colony forming units (CFU/mL) was determined and repeated 7 times. For one of the MRSA isolates, the bacteria recovered after one exposure was significantly higher than the starting cell density, suggesting it had already developed resistance to mupirocin. Over the next six exposures, all 12 isolates showed increased growth following treatment with mupirocin, suggesting a developed resistance to mupirocin. In contrast, over the seven repeated exposures to GS‐1, all 12 MRSA isolates showed a gradual decrease in recovered bacteria (Figure 3C). The difference in CFU/mL between mupirocin and GS‐1 was statistically significant after 3 rounds of exposure. This suggests that with repeated exposures to GS‐1, MRSA becomes increasingly susceptible to GS‐1, rather than developing resistance.

### 3.4 GS‐1 Effectively Cleared an MRSA Infection In Vivo

Systemic tolerability of GS‐1 has been previously demonstrated, where rats were found to tolerate a subcutaneous dose as high as 190.5 mg/kg [17]. To determine the efficacy and safety of GS‐1 at clearing gram‐positive infections in vivo, we tested topical GS‐1 in a rodent model of abraded skin infection. Rats were given abrasions on the skin and infected with different clinical isolates of MRSA, then treated twice daily with either GS‐1 (157.6 mg/mL; 8 rats) or saline (8 rats) for 7 days.

**FIGURE 4:**
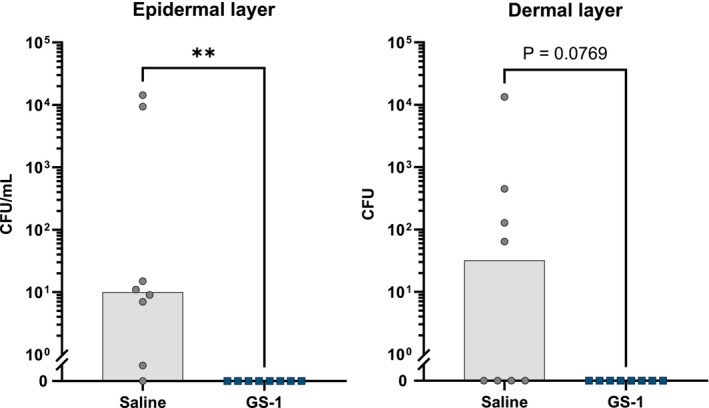
Treatment with GS‐1 significantly reduced topical and dermal MRSA infection in animals. Rats were shaved and skin abraded, then inoculated with 1 × 10^6^ CFU/mL of different clinical isolates of MRSA. After 24 h, rats were treated with either 157.6 mg/mL GS‐1 (8 rats) or saline (8 rats) twice daily for 7 day. On day 7, wounds were imaged, swabbed, and swabs cultured to calculate CFU/mL recovered (epidermal). Tissue punches were taken from the skin of each animal and cultured (dermal) (median, Mann–Whitney *U* test, **p < 0.01).

To understand the effect of GS‐1 treatment on the superficial infection, bacteria were recovered from the epidermal layer of the skin by swabbing the wounds. Of the saline treated animals, 6/8 rats exhibited epidermal infections, and two of these displayed fulminant infections (> 10 000 CFU/mL). The remaining 2/8 rats had ≤ 1 CFU/mL MRSA present in the epidermis. In contrast, all 8/8 GS‐1 treated animals exhibited no detectable bacteria in the epidermis after 7 day of treatment (Figure 4A). This suggests GS‐1 treatment was effective at clearing superficial infection in the epidermis.

To understand whether topical GS‐1 could penetrate deeper into the dermal layers of the skin to clear a deeper bacterial infection, bacteria were recovered from the dermal layers by collecting skin punctures and culturing the tissue. Of the saline‐treated animals, 4/8 rats exhibited MRSA present in the dermis, indicative of a deeper active infection. The other 4/8 rats returned no detectable bacteria from the dermal layer, suggesting they successfully cleared the dermal infection. In contrast, all 8/8 GS‐1‐treated animals exhibited no detectable dermal infection after 7 day of treatment (Figure 4A). This demonstrates that GS‐1 was able to penetrate into the dermal layers of the skin to successfully eradicate MRSA from all infected animals after 7 day of treatment.

To identify any toxic effects of topical GS‐1 treatment, blood was collected from rats at the completion of the study, and haematological and clinical metrics were measured (Table 2). Analysis of blood taken after the completion of the study showed no statistically significant differences between GS‐1‐treated, saline‐treated, or uninfected animals for any haematological or clinical chemistry parameter.

**TABLE 2:** Haematology and clinical chemistry results following topical GS‐1 treatment in rats.

Analyte	Units	GS‐1‐treated mean ± SD	Saline‐treated mean ± SD
White blood cell	×10^3^/μL	4.85 ± 1.78	6.28 ± 3.77
Red Blood cell	×10^6^/μL	8.52 ± 0.95	7.62 ± 1.00
Haemoglobin	gm/dL	14.70 ± 0.88	13.9 ± 1.39
Haematocrit	%	47.40 ± 3.69	42.5 ± 5.74
Platelets	×10^3^/μL	540.00 ± 192.00	610.00 ± 75.40
Na	mEq/L	145.80 ± 2.31	145.50 ± 1.41
K^+^	mEq/L	5.34 ± 0.57	5.28 ± 0.78
Cl^−^	mEq/L	100.30 ± 2.71	101.50 ± 1.93
Glucose	mg/dL	306.80 ± 106.70	233.30 ± 107.90
Blood urea nitrogen	mg/dL	20.00 ± 2.93	19.75 ± 2.43
Creatinine	mg/dL	0.35 ± 0.08	0.29 ± 0.08
Alkaline phosphatase	Units/L	82.88 ± 22.82	101.8 ± 12.89
Aspartate transferase	Units/L	56.00 ± 8.14	60.50 ± 8.40
Alanine transferase	Units/L	30.00 ± 3.30	23.75 ± 13.00
Amylase	Units/L	2209.00 ± 354.90	2250.00 ± 329.20
Creatinine kinase	Units/L	335.60 ± 209.90	295.90 ± 134.00
Cholesterol	mg/dL	81.25 ± 13.37	77.13 ± 13.50
Globulin	mg/dL	1.93 ± 0.13	2.00 ± 0.12
Albumin	mg/dL	3.89 ± 0.18	3.75 ± 0.11
Protein	mg/dL	5.81 ± 0.18	5.75 ± 0.19
Calcium	mg/dL	11.24 ± 0.47	11.18 ± 0.37
Phosphorous	mg/dL	10.41 ± 1.72	10.14 ± 1.20
Total bilirubin	mg/dL	< 0.1	< 0.1
Direct bilirubin	mg/dL	< 0.1	< 0.1

### 3.5 GS‐1 Does Not Induce Irritation or Sensitisation in Human Subjects

To validate the topical safety of GS‐1 in humans, a Repeat Insult Patch Test was conducted in 111 subjects, including 57 subjects with self‐perceived sensitive skin. No dermal reactions were observed during the 3‐week Induction Phase of the study in any of the participants. During the Challenge Phase, 2 subjects out of 111 displayed mild to well‐defined erythema after 48 h, which resolved to barely perceptible erythema or no visible skin reaction by 96 h post‐challenge (Table 3). Individual dermal scores recorded during the Induction and Challenge Phases appear in Table 3 for subjects that elicited dermal reactions, missed a visit, or were discontinued. All other subjects did not exhibit any dermal reactions throughout the course of the entire study and had scores of ‘0’. No adverse events were reported over the duration of the study. These results suggest GS‐1 does not elicit any dermal irritation or clinically significant potential for inducing sensitisation.

**TABLE 3:** GS‐1 dermal scores following an occluded RIPT with GS‐1 (157 mg/mL) in human subjects. All other 100 subjects except those listed below did not exhibit any dermal reactions throughout the course of the entire study and had scores of ‘0’.

Subject number	Induction phase scores									Challenge scores			
	1	2	3	4	5	6	7	8	9	24 h	48 h	72 h	96 h
8	0	0	0	0	0	0	0	0	0	0	0	0	—
16	0	0	0	0	0	Discontinued							
24	0	0	0	0	0	0	Discontinued						
27	0	0	0	0	0	0	0	0	0	0	0	X	0
33	0	Discontinued											
34	0	0	0	0	0	0	0	0	0	0	0	X	0
44	0	0	0	0	0	0	0	0	0	X	X	0	0
50	0	0	0	0	0	0	0	0	0	0	0	0	X
58	0	0	0	0	0	0	0	0	0	0	2+	1+	±±
66	0	0	0	0	0	0	0	0	0	0	1+	0	0
75	0	Discontinued											
86	0	0	0	0	Discontinued								
90	0	0	0	0	0	0	0	0	0	0	0	X	0
101	0	0	0	0	0	0	0	0	0	Discontinued			
104	0	0	0	0	0	0	0	0	0	0	0	0	—
107	0	0	0	0	Discontinued								
108	0	Discontinued											
116	Discontinued												
119	0	0	0	0	0	0	0	0	0	0	0	0	—
120	0	0	0	0	0	0	0	0	0	0	0	0	—

Abbreviations: —, no reading; ±, barely perceptible erythema; 0, no visible skin reaction; 1+, mild erythema; 2+, well defined erythema; X, subject absent.

## 4 Discussion

Fatty acids have been studied as potential antimicrobial agents for over 100 years. However, with the advent of modern antibiotics in the 1930s, much of this research has ceased. With the development of antibiotic resistance, there was a renewed interest in fatty acid compounds in the 1970s and 1980s. However, the difficulty in working with highly hydrophobic compounds made practical applications as antimicrobials difficult. Undecylenic acid is an organic, unsaturated fatty acid produced by ‘cracking’ castor oil under high pressure [18, 19, 20, 21]. Undecylenic acid is currently FDA‐approved for over‐the‐counter use to treat fungal infections of the skin. However, it is not commonly used as the development of newer antifungal agents such as imidazoles and triazoles has garnered a larger share of the antifungal market. In this study, we present evidence that a water‐soluble ammonium carboxylate salt of undecylenic acid and L‐arginine, GS‐1, was effective against the causative agents of impetigo in vitro and in vivo.

GS‐1 is manufactured via a simple process of combining undecylenic acid, arginine, and water to form stable micelles, a process completed in hours and requiring only basic equipment, with a shelf‐life of at least 24 months. In contrast, the production of mupirocin involves a fermentation process with *Pseudomonas fluorescens* , followed by complex extraction and purification requiring specialised equipment and materials, with a total production time of several days. The simple method of manufacturing GS‐1 and its long shelf‐life make it economical to produce at scale, offering a practical and cost‐effective alternative to current impetigo treatments.

The data presented in this study clearly demonstrates that GS‐1 can achieve a bactericidal effect against common gram‐positive cocci skin pathogens. The concentration difference between the MIC and MBC was relatively small (2–3×) with several strains sharing the same concentration for inhibition as well as bactericidal activity. This observation suggests a ‘threshold effect’ whereby the effective mechanism of action requires that a set concentration, regardless of the strain, be achieved before activity is observed. However, once this threshold concentration is achieved, large increases in concentration do not significantly alter the effect.

Based on the MIC and MBC data, the concentrations of GS‐1 required to kill 99.99% MRSA under CLSI conditions is significantly higher at 1.26 mg/mL than intravenous vancomycin, which is 2–4 μg/mL [22]. Despite this, the low toxicity risk of fatty acids allows GS‐1 to be used at much higher concentrations than could be achieved safely with standard antibiotics such as vancomycin. As a potential impetigo treatment, GS‐1 could be dosed topically at concentrations far exceeding the MBC to ensure efficacy, whilst still maintaining safety. Indeed, we have previously reported GS‐1 to be well tolerated in rats at 190 mg/kg when administered subcutaneously [17]. Furthermore, findings from the RIPT in human subjects reported here also support the safety and tolerability rationale of topical GS‐1. This study revealed that GS‐1 elicits antibacterial activity by permeabilizing bacterial membranes and inducing ROS production. The opposing charges in GS‐1 and bacterial cell membranes may facilitate the binding of GS‐1 micelles to the bacterial surface to elicit structural changes and membrane disruption. Based on previous research and our own findings, it is possible that the GS‐1 supramolecular structure is interfering with wall teichoic acids (WTAs) in the cell walls of gram‐positive bacteria. WTAs are phosphate‐rich, sugar‐based polymers attached to the cell walls of many gram‐positive bacteria [23, 24, 25]. These anionic polymers serve to reduce osmotic stress as well as regulate cell division, mediate host colonisation, and protect enzymatically susceptible peptidoglycan bonds [25]. Given the relatively large cationic charge across the surface of the GS‐1 supramolecular structure, it is plausible that GS‐1 binds to anionic WTAs, leading to a defect in the cell wall that is the first step in compromising the viability of the bacteria.

Antibacterial resistance is emerging as a significant threat to global health, making it critical to understand if and how novel antibiotics might induce resistance. This study found that MRSA did not appear to develop resistance to GS‐1 over 25 exposures. Rather, we observed a slight decrease in viability with repeated exposures. It is possible that exposing the bacteria at sub‐MIC doses introduced GS‐1 into the anionic WTAs present in the cell wall, either bound to the surface or incorporated into the bacterial membrane or cell wall. These bound particles of GS‐1 may remain as part of the membrane, which could gradually reduce viability. As the bacteria divide, the daughter cells may carry the defect, and with repeated exposures to GS‐1, the number of bound GS‐1 particles increases. Once a critical threshold of binding occurs, the bacteria may lose viability and become increasingly susceptible to GS‐1. This may explain why MRSA previously exposed to GS‐1 showed approximately 10 times less growth than naïve MRSA following the final GS‐1 exposure.

Findings from this study suggest that repetitive exposure of MRSA to GS‐1 may not induce resistance mechanisms, as is observed with mupirocin. Interestingly, both GS‐1 (undecylenic acid) and mupirocin are medium‐chain fatty acids (mupirocin is a fatty acyl). Despite this shared chemical taxonomy of mupirocin and GS‐1, there are key differences in their structures and mechanisms of action that may explain the different responses observed following repeated exposures. Mupirocin works by inhibiting bacterial protein synthesis, targeting the bacterial enzyme isoleucyl‐tRNA synthetase, which is essential for incorporating the amino acid isoleucine into proteins during translation [26]. GS‐1, in contrast, appears to act directly on bacterial cell walls and membranes, rapidly causing membrane rupture and bacterial cell death. These differences in mechanisms of action between mupirocin and GS‐1 may be attributable to their different chemical structures and could help explain the different responses in MRSA following multiple exposures to either drug.

Resistance to mupirocin in *S. aureus* occurs mainly through two mechanisms. Low‐level resistance arises from point mutations in the ileS gene, which encodes the isoleucyl‐tRNA synthetase, reducing the antibiotic's binding affinity. High‐level resistance is typically due to the acquisition of a plasmid carrying the mupA or mupB gene, which encodes a modified isoleucyl‐tRNA synthetase that mupirocin cannot inhibit [26]. Since it appears that GS‐1 directly targets bacterial cell membranes, acting similarly to a surfactant through its supramolecular structure, it is possible that bacteria would find it difficult to develop a mechanism to become resistant. Though further work is needed to fully understand the effects of repeated exposure to GS‐1 on bacteria, results from this study show promise that repeated exposures to GS‐1 are less likely to induce antibiotic resistance.

While there are differences in potency between GS‐1 and mupirocin, GS‐1 presents a viable alternative impetigo treatment. Despite mupirocin demonstrating superior MICs at < 0.5 μg/mL [27], compared to GS‐1's 0.63–1.26 mg/mL, GS‐1 supersedes mupirocin in bactericidal efficacy, with MBCs at 1–4× its MIC, whereas mupirocin requires concentrations 8–32× higher than its MIC for bactericidal action [27]. Time‐kill studies show GS‐1 achieves a 2‐log reduction in bacterial load within 2 h and a sustained 2–3‐log reduction within 4–6 h, comparable to mupirocin's reduction over 24 h [27]. Critically, GS‐1 demonstrates no resistance development after repeated exposure, a key advantage over mupirocin, which faces rising resistance rates exceeding 10% in high‐use areas [5]. This positions GS‐1 as a compelling alternative to mupirocin for treating impetigo.

The in vitro antibacterial efficacy of GS‐1 translated to in vivo experiments. Our studies demonstrate that GS‐1 successfully treated MRSA skin infection in rodents. To maximise clinical relevance, rats were challenged with different clinical isolates of MRSA, making the task even more challenging. Not only did GS‐1 clear the superficial infection present in the epidermal layer of the skin, but it was also able to penetrate deep into the skin to kill bacteria that had colonised within the dermis. It is important to note that the rat epidermis is notably thinner than in humans. While the lack of bacteria recovered from the dermis of GS‐1‐treated rats, as shown here, suggests GS‐1 can permeate into the dermis, it is unclear whether this would occur during human dermal infections, where the significantly thicker epidermis presents a greater barrier. Future studies employing human skin models, such as in vitro permeation testing and clinical trials, will be critical to validating these results and ensuring GS‐1's therapeutic potential translates to human skin infections.

It is worth noting that several of the saline‐treated animals cleared their infection without any treatment. This observation was not unexpected, given that rodents can spontaneously clear topical infections without treatment. Even after allowing for the spontaneous clearance in the control group, the observation that all GS‐1‐treated animals successfully eradicated the MRSA infection following treatment still suggests that topical GS‐1 produced a measurable antibacterial effect in the infected skin. Further to this, the observation that GS‐1‐treated animals displayed no difference in clinical chemistry and haematology markers to saline‐treated and untreated rats further supports the safety rationale for topical GS‐1.

Interestingly, GS‐1 also demonstrated inhibitory activity against gram‐negative bacterial pathogens. However, the MICs observed for gram‐negative bacteria were higher than those of gram‐positive bacteria; therefore, GS‐1's enhanced efficacy against gram‐positive pathogens makes it particularly suitable for infections caused by these bacteria, such as impetigo. Additionally, GS‐1's broad‐spectrum activity extends beyond bacteria, with demonstrated activity against fungal pathogens. GS‐1's activity against fungi is not surprising, given that undecylenic acid is currently used to treat fungal infections. Nevertheless, the expanded activity of GS‐1 against bacterial pathogens shown here indicates GS‐1's potential as a broad‐spectrum antimicrobial agent.

In this study, we have presented evidence that a novel ammonium carboxylate salt of undecylenic acid and L‐arginine, GS‐1, produced a potent antibacterial effect both in vitro and in vivo against the gram‐positive coccobacilli responsible for common skin and soft tissue infections such as impetigo. We demonstrated that GS‐1 acts on bacterial cell membranes to induce bactericidal activity. No evidence of resistance against GS‐1 was observed across multiple exposures to GS‐1. Finally, topical GS‐1 demonstrated efficacy at clearing MRSA infection in vivo with no signs of toxicity associated with the treatment, and repeated skin exposure to GS‐1 in human subjects was well tolerated. Based on these findings, we propose that GS‐1 may have significant potential as a topical treatment for uncomplicated skin infections involving MRSA, MSSA, and *S. pyogenes* .

## Author Contributions

Conceptualisation: Thomas Rau. Data curation: Thomas Rau and Alyce Mayfosh. Formal analysis: Thomas Rau and Alyce Mayfosh. Methodology: Thomas Rau. Project administration: Alyce Mayfosh. Writing – original draft: Thomas Rau. Writing – review and editing: Alyce Mayfosh. All authors have read and agreed to the published version of the manuscript.

## Ethics Statement

The animal study protocol was approved by the Animal Ethics Committee of the University of Montana Institutional Animal Care and Use Committee, 052‐18TRPC‐103 118; approved 31 October 2018.

## Conflicts of Interest

A.M. and T.R. are employees of Wintermute Biomedical.

## Patents

US patent 11 103 475; Issue date 31 August 2021. Therapeutic Compositions of Undecylenic Acid and Arginine.

## Supporting information


Data S1.

## Data Availability

The data that support the findings of this study are available from the corresponding author upon reasonable request.
