# Supplementary material for: A Novel Ammonium Carboxylate Salt of Undecylenic Acid for the Topical Treatment of Gram‐Positive and Antibiotic‐Resistant Skin Infections
Source: Exp Dermatol. 2025 Mar 10;34(3):e70075. doi: 10.1111/exd.70075 (PMC11891955; doi:10.1111/exd.70075)
Supplement: Supplementary file 1 — Data S1. [file EXD-34-e70075-s001.docx]

# Supplementary Material

***Stability testing***

## Physical and Chemical Testing

The physical characteristics of GS-1 were assessed by visually examining the product in a clear container, recording its appearance and detecting any foreign matter. pH measurements were performed using a calibrated pH meter at 23–25°C according to USP <791>/Ph Eur 2.2.3 standards, with an acceptance range of 8.3 ± 0.5.

## Assay and Related Substances Analysis

Quantification of undecylenic acid and its impurities was performed using HPLC-UV (Agilent Infinity 1260 or equivalent) with a Phenomenex Prodigy ODS-3 column (250 × 4.6 mm, 5 µm). The mobile phase consisted of 50% acetonitrile with 0.1% phosphoric acid in water, filtered through a 0.2 µm membrane. Samples and standards were prepared in duplicate, with calibration and validation for linearity (0.1–130% of nominal concentration), precision (70–130%), and accuracy. Acceptance criteria were 90.0–110.0% w/w of label claim for each undecylenic acid and arginine. Related substances were quantified with a reporting threshold of 0.05%.

**Supplementary Table 1. Stability of GS-1 over 12 months at real-time and accelerated conditions.**

| **Condition** | **Time Point (Months)** | **Undecylenic Acid Assay (%)** | **Undecylenic Acid Related Substance Total (%LC)** | **Arginine Assay (%)** | **Arginine Related Substance Total (%LC)** | **pH** | **Appearance** |
| --- | --- | --- | --- | --- | --- | --- | --- |
| **25°C/60% RH** | 0 | 100 | 3.8 | 102 | 0 | 8.4 | Clear, light yellow, free from foreign matter, slightly viscous |
| **25°C/60% RH** | 1 | 101 | 5.4 | 101 | 0.1 | 8.4 | Clear, light yellow, free from foreign matter, slightly viscous |
| **25°C/60% RH** | 6 | 101 | 5.3 | 103 | 0.2 | 8.4 | Clear, light yellow, free from foreign matter, slightly viscous |
| **25°C/60% RH** | 12 | 102 | 4.4 | 104 | 0.3 | 8.6 | Clear, yellow, free from foreign matter, slightly viscous |
| **40°C/75% RH** | 1 | 102 | 5.2 | 101 | 0.1 | 8.4 | Clear, light yellow (slightly darker than 25°C/60% RH), free from foreign matter, slightly viscous |
| **40°C/75% RH** | 3 | 101 | 4.8 | 103 | 0.3 | 8.4 | Clear, light yellow, free from foreign matter, slightly viscous |
| **40°C/75% RH** | 6 | 102 | 4.0 | 104 | 0.6 | 8.4 | Clear, dark yellow, free from foreign matter, slightly viscous |

LC, Label Claim; RH, Relative Humidity.

***Determination of MIC against gram-negative bacteria and fungi***

Wintermute Biomedical utilized the non-clinical and pre-clinical services program offered by the National Institute of Allergy and Infectious Diseases to complete this testing.

Bacterial identifications were confirmed by Element Iowa City (JMI Laboratories) using matrix-assisted laser desorption ionization-time of flight mass spectrometry (Bruker Daltonics, Bremen, Germany). Bacterial isolates were tested for antimicrobial susceptibility using broth microdilution methodology according to Clinical and Laboratory Standards Institute (CLSI) M07 (2024) and M100 (2024) guidelines. Minimal Inhibitory Concentration (MIC) testing was conducted in Tryptic Soy broth (TSB). The inoculum density target of 5 x 10^5^ CFU/mL (5 x 10^4^ CFU/well) during susceptibility testing was monitored by bacterial colony counts.

Fungal MICs were measured according to CLSI M27 & M38 guidelines. All testing was performed in RPMI buffered with 0.165M MOPS. MICs were read after 24–72 h at 50% and 100% inhibition of growth.

**Supplementary Table 2. MIC of GS-1 against gram-negative pathogens.**

| **Organism** | **Collection No.** | **MIC (mg/mL)** |
| --- | --- | --- |
| *Acinetobacter baumannii* | NCTC 13304 | 4.096 |
| *Acinetobacter baumannii-calcoaceticus* species complex | 1270289 | 4.096 |
| *Acinetobacter baumannii-calcoaceticus* species complex | 1281556 | 4.096 |
| *Enterobacter cloacae* species complex | 1276012 | 8.192 |
| *Enterobacter cloacae* species complex | 1277814 | >8.192 |
| *Enterobacter cloacae* species complex | 1304909 | >8.192 |
| *Enterococcus faecalis* | ATCC 29212 | 2.048 |
| *Enterococcus faecalis* | 1271850 | 2.048 |
| *Enterococcus faecium* | 1279093 | 2.048 |
| *Escherichia coli* | ATCC 25922 | 8.192 |
| *Escherichia coli* | 1273672 | 8.192 |
| *Escherichia coli* | 1281539 | 8.192 |
| *Klebsiella pneumoniae* | 1253428 | 8.192 |
| *Klebsiella pneumoniae* | 1278251 | >8.192 |
| *Klebsiella pneumoniae* | 1281849 | >8.192 |
| *Proteus mirabilis* | 1233970 | 8.192 |
| *Proteus mirabilis* | 1248529 | >8.192 |
| *Proteus mirabilis* | 1293712 | >8.192 |
| *Pseudomonas aeruginosa* | ATCC 27853 | 8.192 |
| *Pseudomonas aeruginosa* | 1296381 | 8.192 |
| *Pseudomonas aeruginosa* | 1245277 | 4.096 |

**Supplementary Table 3. MIC_50_ and MIC_100_ of GS-1 against fungal pathogens.**

| **Species** | **Isolate No.** | **GS-1 (mg/mL)** | |
| --- | --- | --- | --- |
|  |  | **MIC_50_** | **MIC_100_** |
| ***Yeasts*** | | | |
| ***Candida parapsilosis*** | ATCC 22019 | 0.25 | 1.96 |
| ***Candida krusei*** | ATCC 6258 | 0.25 | 0.49 |
| ***Candida albicans*** | SC5314 | 0.25 | 0.49 |
|  | CA90028 | 0.25 | 0.49 |
|  | CA1 | 0.125 | 0.25 |
| ***Candida auris*** | DI17-47 | 0.125 | 0.25 |
|  | DI17-48 | 0.125 | 0.49 |
|  | DI17-46 | 0.25 | 0.49 |
| ***Candida glabrata*** | CG3 | 0.25 | 0.49 |
|  | 05-62 | 0.06 | 0.25 |
|  | 05-761 | 0.25 | 0.49 |
| ***Cryptococcus neoformans*** | USC1597 | ≤0.03 | 0.06 |
|  | H99 | ≤0.03 | 0.125 |
|  | DI19-14 | ≤0.03 | ≤0.03 |
| ***Molds*** | | | |
| ***P. variotii*** | MYA-3630 | 0.25 | 0.49 |
| ***Aspergillus fumigatus*** | AF293 | 0.49 | 0.98 |
|  | DI15-106 | 0.49 | 0.98 |
|  | DI15-116 | 0.49 | 0.98 |
| ***Mucor* spp.** | Mucor1 | 0.125 | 0.49 |
|  | Mucor2 | 0.125 | 0.49 |
|  | Mucor3 | 0.125 | 0.49 |
| ***Blastomyces dermatitidis*** | BD1 | ≤0.03 | ≤0.03 |
|  | BD2 | ≤0.03 | 0.06 |
|  | BD3 | ≤0.03 | 0.06 |
